# High‐density mapping of multiple atypical atrial flutter. First HD GRID mapping experience among The Commonwealth of Independent States

**DOI:** 10.1002/joa3.12384

**Published:** 2020-06-16

**Authors:** Abay Bakytzhanuly, Ayan Abdrakhmanov, Telman Seisembekov, Aliya Smagulova, Dariga Blyalova

**Affiliations:** ^1^ Interventional Arrhythmology Department JSC “National Research Cardiac Surgery Center” Nur‐Sultan Kazakhstan; ^2^ Cardiology Department JSC “Astana Medical University Nur‐Sultan Kazakhstan

**Keywords:** atrial tachycardia, atypical atrial flutter, directional high‐density mapping catheter, HD Grid catheter, high‐density mapping

## Abstract

Mapping of multiple atrial tachycardias after previous cryoballoon pulmonary vein isolations and multiple radiofrequency ablations can be challenging even for experienced specialists. HD Grid high‐density mapping catheter is one of the catheters, which helps not only to precisely identify the mechanisms of macro‐reentry tachycardia but also to avoid unnecessary radiofrequency applications. Accordingly, we present two cases of complex atrial arrhythmia with the use of HD Grid, which showed clear visualization of mechanisms and target ablations with the termination of tachycardia.

## Graphical abstract

In our article, we describe two clinical cases with multiple atrial arrhythmias in patients who underwent previous catheter ablation (cryoablation, multiple). It is the first experience among CIS in using high‐density mapping catheter HDGRID.

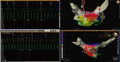

## 1 INTRODUCTION

Pulmonary veins isolation (PVI) forms the cornerstone of catheter ablation for atrial fibrillation (AFib).^1^ Often PVI is followed by linear applications when atrial flutter (AFL) is registered.^2^ Resumption of AFib often occurs due to reconnection of PVs with the left atrium (LA) and becomes a substrate for the development of other atrial arrhythmias, such as atypical AFL. The latter one requires precise localization of the reconnection spot, identifying other ectopic sources, and determining the mechanisms of atypical AFL.^3^ A circular multipolar catheter frequently used to identify the exit and entrance block during PVI. Thus, the use of these circular catheters increases the PVI efficiency comparatively with the use of only one ablation catheter. However, in some cases, the complexity of PVs anatomy in relation to the LA remains underestimated and leads to unnecessary extra applications than is actually required.^4^

In relation to this problem, HD Grid mapping catheter (Abbott Technologies, Minneapolis, MN) was invented. The advantage of this electrode is the ability to perform quickly and precise mapping with 16 poles, which has the same distance between each other. The above characteristics make it possible to perform substrate mapping with rather high accuracy in comparison with other linear diagnostic catheters.^5^

In this article, we describe two clinical cases of high‐density mapping with HD Grid catheter. Due to a prolonged arrhythmic history, patients took rivaroxaban 20 mg for at least 3 months before the current ablation. In both cases, an electroanatomical, bipolar and pace mappings were performed via Ensite Precision 3D mapping system (Abbott Technologies, Minneapolis, MN).

## 2 CLINICAL CASE 1

A 69‐year‐old man was presented with a 2‐year history cryoballoon isolation of PVs. Due to symptomatic and ineffective anti‐arrhythmic therapy, catheter ablation was decided to be performed. The index procedure was performed under moderate sedation and local anesthesia with a standard femoral vein approach. A decapolar catheter (Inquiry 6 French decapolar catheter [Abbott Technologies]) was placed at coronary sinus (CS) and ablation catheter (Therapy 6 French quadripolar catheter [Abbott Technologies]) was placed in the right atrium. Standard transseptal puncture (TSP) was performed to check the sustained PVI. HD Grid catheter was used to create a bipolar map on sinus rhythm with a peak‐to‐peak bipolar voltage 0.2‐04 mV. Thus, we identified the absence of left PVI. Performed re‐isolation of left PVs with the power of 30V on posterior and inferior walls, 35V on superior and anterior walls. Left PVs re‐isolation followed by burst stimulations with circle length (CL) 250 milliseconds, which induced AFL with the earliest activation of proximal CS electrodes with a tachycardia CL of 257 milliseconds. Series of entrainment revealed no early activation zones in the LA but proximal CS post‐pacing interval (PPI) was the earliest with 295 milliseconds. Cavotricuspid isthmus (CTI) was entrained by the ablation catheter (PPI‐275 milliseconds), which confirmed the isthmus‐dependent AFL mechanism. Radiofrequency ablation (RFA) of CTI followed by bidirectional isthmus block.

## 3 CLINICAL CASE 2

**FIGURE 1:**
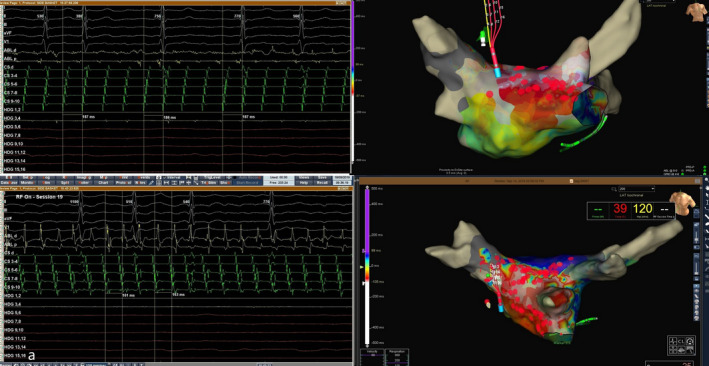
RFA of atypical left AFL. After the confirmation of sustained PVI, an atypical left AFL was induced by burst stimulations. CS showed proximal electrodes activation with CL 187 ms. Entrainment and activation mapping identified the perimitral mechanism of atypical AFL. Anterior linear RFA leads to the tachycardia CL change and the earliest activation of the distal CS electrodes. Additional roof line lead to the tachycardia termination and induction of other tachycardia

**FIGURE 2:**
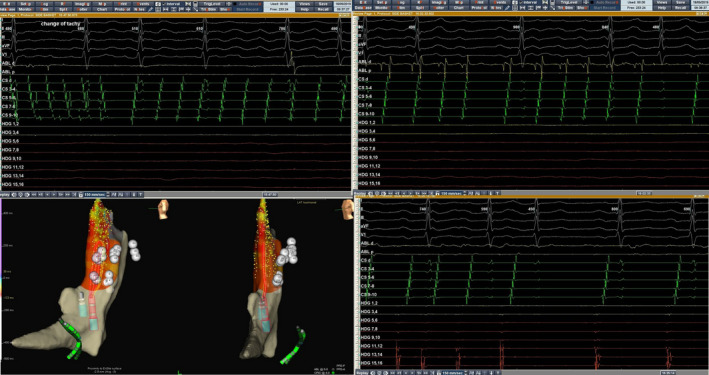
RFA of SVC. F‐waves morphology on ECG during tachycardia and P‐waves during sinus rhythm is similar. Activation mapping with HD Grid of SVC during atrial tachycardia identified the earliest zones in SVC. RFA from SVC up to high right atrium (without SVC isolation) terminated the tachycardia. Local activation time was −119 ms. Activation map projections: left lateral and left anterior oblique

A 57‐year‐old man was referred to our hospital because of symptomatic drug‐refractory atrial arrhythmia. The patient had undergone cryoballoon PVI, radiofrequency PVs’ re‐isolation, linear RFA of mitral isthmus during the last 5 years. We used the same approach and catheters mentioned above. Bipolar mapping of LA showed sustained PVI. Atypical AFL with CL 187ms was induced by burst stimulations. The earliest activation was on proximal CS electrodes. For the first sight, it looked like a typical AFL as in the first clinical case, but entrainment excluded right atrial arrhythmia. After TSP and LA activation mapping, we identified perimitral atypical AFL which was entrained and confirmed. Linear RFA from the mitral valve to the right superior PV was performed (parameters of ablation: P‐35V, irrigation 30 mL/s, impedance 103‐118 Om, t‐ 48°C). When the block was achieved the tachycardia CL (163 milliseconds) and activation on CS electrodes changed. Additional linear RFA of the roof performed with similar ablation parameters (Figure 1), which lead to the tachycardia termination and induction of other tachycardia. If we pay attention to F‐wave morphology on the ECG during tachycardia and compare it with P‐wave during sinus rhythm, we can identify the similarities. Thus, we assumed, that ectopic focus localized around the sinus node. Activation mapping with HD Grid of SVC during atrial tachycardia identified the earliest zones in SVC (Figure 2). The local activation time was −119 milliseconds. RFA from SVC up to high right atrium (without SVC isolation) terminated the tachycardia.

## 4 DISCUSSION

In patients with a history of cardiac surgery procedures and multiple catheter ablation mechanisms of arrhythmias suggested to be complicated. Our cases demonstrate the use of a HD mapping catheter to facilitate the identification of mechanisms of tachycardia. The HD Grid is a rectangular shape HD mapping catheter consists of 16 electrodes that were equally distributed across four splines (four electrodes 3 mm electrode per spline with an inter‐electrode distance of 3 mm). Our cases demonstrate the first experience of HD GRID mapping among the Commonwealth of Independent States.

## 5 CONCLUSION

The HD Grid mapping catheter allows not only rapid assessment of voltage and activation, but also to facilitate the identification of complex atrial arrhythmias as atypical AFL. Especially when patients after cardiac interventions which leads to scar formations and multiple scar‐related atrial arrhythmias.

## CONFLICT OF INTEREST

No conflict of interest. First author and all co‐authors are physicians with no connection to Abbott.
